# Patterns of Drug and Polydrug Detection in Drivers Suspected of Driving Under the Influence of an Intoxicant in Ireland 2019–2020: A Latent Class Analysis

**DOI:** 10.1111/dar.70087

**Published:** 2025-12-14

**Authors:** Louise Durand, Aoife O'Kane, Richard Maguire, Denis Cusack, Eamon Keenan, Gráinne Cousins

**Affiliations:** ^1^ School of Pharmacy and Biomolecular Sciences RCSI University of Medicine and Health Sciences Dublin Ireland; ^2^ Medical Bureau of Road Safety, Health Science Centre University College Dublin Dublin Ireland; ^3^ National Social Inclusion Office, Health Service Executive Dublin Ireland

**Keywords:** amphetamines, benzodiazepines, cannabis, cocaine, opioids, road safety, toxicological analysis

## Abstract

**Introduction:**

Driving under the influence of drugs is a major risk factor for road traffic collisions. While increasing harms are observed in relation to polydrug use, evidence is needed about this issue in the context of road safety. We examined polydrug use patterns in drivers providing samples for toxicological analysis in Ireland between 2019 and 2020.

**Methods:**

A cross‐sectional study using LC–MS toxicology results from the Medical Bureau of Road Safety, which is responsible for the chemical testing of intoxicants in all drivers arrested under the Road Traffic Acts 1968–2024 in Ireland. Latent class analysis was performed on all samples with at least one drug detected (*N* = 4856). Descriptive statistics for age, gender and number of drug groups detected were calculated for each class identified.

**Results:**

We identified six latent classes based on drug detection patterns. The cannabis only class (46.5%) is characterised by the detection of cannabis with no other drug involved, a high proportion of men and young age. The cocaine class (31.1%), which combines cocaine and cannabis use, and the stimulant class (2.5%), characterised by amphetamine/methamphetamine detection, have a similar demographic profile to the cannabis class. The polydrug non‐opioid (11.8%), polydrug opioid (5.5%) and heroin (2.6%) classes are older, with lower male:female ratios.

**Discussion and Conclusions:**

By identifying profiles of people driving under the influence of drugs, this study contributes to enhancing knowledge of drug and polydrug use in motor vehicle drivers in Ireland. Further work is needed to examine risks and develop interventions to address polydrug driving.

## 1 Introduction

Road traffic collisions (RTC) represent the leading cause of death for children and young adults aged 5–29 years, and are the 12th leading cause of death across all ages, with 1.19 million road traffic deaths in 2021 [1]. In addition, RTC is a leading cause of global disability‐adjusted life years, resulting in a significant economic and social burden [2]. Vehicle safety features and road infrastructure advancements along with the implementation of safer road policies have led to significant progress and declines in road traffic deaths in many high‐income countries, including Ireland, since the early 1990s [2]. However, progress has appeared to slow and plateau between 2013 and 2021, and a 38% increase in RTC‐associated deaths was reported between 2021 and 2023 [3].

Driving under the influence of psychoactive substances is associated with RTC and fatalities [4]. Driving is a high‐performance divided attention task requiring sustained engagement, continuous information processing and motor skills to adapt to dynamic road and traffic conditions, as well as behavioural and emotional control. While alcohol is the psychoactive substance most frequently identified as associated with RTC [1], an increased risk of RTC is also observed with the use of a number of psychoactive drugs. These include cannabis [5], cocaine [6], amphetamines and methamphetamines [6], benzodiazepines [6], opioids [6] and gabapentinoids [7] independently.

However, evidence shows that drugs are commonly consumed in combination [8, 9], including in impaired vehicle drivers [10, 11, 12]. For example, additive or synergistic effects can be obtained, such as by combining benzodiazepines [13] or gabapentinoids [14] with opioids. Management of drug effects can also be sought, for example, by pairing cocaine with cannabis [15]. Therefore, reporting drugs independently may result in attributing the effect of a drug or drug combination to another due to a confounding effect. Challenges exist and warrant more research in quantifying the impairment associated with the co‐use of several drugs as it is dependent on multiple factors, including the individual, the drugs consumed, their dosage and sequence of use [16]. Nevertheless, polydrug use (i.e., using more than one drug at the same time or within a short period of time) in drivers was found to be associated with increased harms compared to single drug use, including RTC involvement, injury [17], culpability [16] and death [18].

In previous work, the authors of this study have identified increasing trends in the detection of cocaine and cannabis as well as the co‐detection of cocaine with cannabis, and cocaine with benzodiazepines in drivers suspected of driving under the influence of drugs in Ireland between 2012 and 2018 [10]. As preferential drug combinations are observed within different populations [19], identifying groups most likely to consume certain substances, alone or in combination has the potential to further inform the scope and target of potential interventions.

Latent Class Analysis (LCA) is a statistical approach that can identify unobserved or latent classes of related cases based on patterns of observed responses to categorical variables [20]. This method has been used to explore polysubstance use patterns in several populations including the general population [19], people who use drugs [21], overdose deaths [22] and vehicle drivers [23, 24, 25]. Prior LCA studies in road users had limitations for generalisability, such as the restriction of the study sample to hallucinogen drug users [23], or samples with two or more substances detected [24, 25], therefore masking single drug use patterns and limiting the broader applicability of the findings. Given the potential of LCA to identify distinct subgroups within heterogeneous populations, it appears relevant to apply it to a more inclusive sample of drivers with positive drug toxicology results, without excluding single drug use.

The aims of this study are: (i) to identify drug and poly‐drug use patterns in drivers providing samples for toxicological analysis using LCA; and (ii) to examine demographic differences between identified classes.

## 2 Methods

### 2.1 Design

This is a cross‐sectional study of toxicological results from people suspected of driving under the influence of drugs in Ireland between January 2019 and December 2020. The study is reported according to the Strengthening the Reporting of Observational Studies in Epidemiology (STROBE) guidelines, REporting of studies Conducted using Observational Routinely‐collected health Data (RECORD) statement [26, 27] (Appendix S1).

### 2.2 Data Source

The Medical Bureau of Road Safety is the statutory body responsible for the chemical testing of intoxicants (alcohol and drugs) in drivers arrested under the *Road Traffic Act*. A biological sample (blood or urine) is collected from drivers arrested for suspicion of impairment/intoxication or testing positive for cannabis, cocaine or opioids in an oral fluid preliminary drug test (PDT). A negative PDT does not rule out toxicological analysis if the Gardaí (Irish law enforcement officers) form the opinion that the driver is intoxicated. All toxicological analyses required under the *Road Traffic Act* between 2019 and 2020 have been conducted by the Medical Bureau of Road Safety. All samples below a given threshold for alcohol (87 mg/100 mL in blood, 115 mg/100 mL in urine in 2019 and 100 mg/100 mL in blood, 135 mg/100 mL in urine in 2020), or by request from the Gardaí, are subject to toxicological screening. Due to this important selection bias, we excluded alcohol from the scope of the analyses. Since 2018, the drug screening of biological samples is conducted using liquid chromatography–mass spectrometry (LC–MS) techniques. Sample type (blood/urine), gender and year of birth are recorded along with toxicology results.

The scope of the LC–MS screening with cut‐off values is presented in Table 1. Quantitative LC–MS results were recoded into binary responses (0 = not detected/below cut‐off, 1 = detected/above cut‐off), and further collapsed into the following drug groups: amphetamine, alprazolam, new psychoactive substances (NPS) benzodiazepine, other benzodiazepine, cannabis, cocaine, gabapentinoids, ketamine, methamphetamine, heroin, codeine, methadone, other opioid, other sedative medications with potential to cause impairment, z‐drug. Alprazolam was separated from other prescription benzodiazepines due to its relatively high abuse potential [28], high prevalence [29] and a metabolic pathway allowing for reliable identification. Cathinones and psychostimulants are within the scope of the LC–MS screening but were not included in the analyses because of low or zero prevalence. The number of drug groups detected was calculated for each sample. The age at the time of sample collection was coded into classes: 16–24, 25–34, 35–44, ≥ 45 years.

**TABLE 1:** Scope of LCMS screening tests, detection cut‐off values and drug groups.

Drug group	Analytes screened	Blood cut‐off (ng/mL)	Urine cut‐off (ng/mL)
Cannabis	HU‐210	10	50
	JWH‐018	10	50
	THC	5	20
	AM‐2201	10	50
	THCA	5	20
Cocaine	Benzoylecgonine	50	100
	Cocaethylene	10	50
	Cocaine	10	100
Amphetamine	Fenfluramine	10	50
	Methylenedioxyamphetamine	20	100
	Methylenedioxyethylamphetamine	20	200
	S‐Amphetamine	20	100
Methamphetamine	Methylenedioxymethylamphetamine	20	100
	S‐Methamphetamine	20	100
Ketamine	Ketamine	20	200
	Norketamine	20	200
Alprazolam	Alpha‐Hydroxyalprazolam	10	50
	Alprazolam	10	50
NPS benzodiazepine	Adinazolam	10	50
	Demoxepam	10	50
	Estazolam	10	50
	Etizolam	10	50
	Flualprazolam	10	50
	Flubromazepam	10	50
	Flubromazolam	10	50
	Phenazepam	10	50
Other benzodiazepine	7‐Aminoclonazepam	10	50
	7‐Aminoflunitrazepam	10	50
	7‐Aminonitrazepam	10	50
	Bromazepam	10	50
	Chlordiazepoxide	10	50
	Clobazam	10	50
	Clonazepam	10	50
	Desalkylflurazepam	10	50
	Diazepam	20	50
	Flunitrazepam	10	50
	Flurazepam	10	50
	Lorazepam	10	50
	Lormetazepam	10	50
	Midazolam	10	50
	N‐Desmethylflunitrazepam	10	50
	Nitrazepam	10	50
	Nordiazepam	50	50
	Oxazepam	50	50
	Prazepam	10	50
	Temazepam	20	50
	Triazolam	10	50
Z‐drug	N‐Desmethylzopiclone	10	50
	Zaleplon	10	50
	Zolpidem	10	50
	Zopiclone	10	50
	Zopiclone‐*N*‐oxide	10	50
Methadone	EDDP	10	100
	Methadone	50	100
Heroin	6‐Acetylmorphine	5	100
	Morphine (without codeine present)	10	100
Codeine	Codeine	10	100
Other opioid	Dihydrocodeine	10	50
	Fentanyl	10	50
	Hydrocodone	10	50
	Norfentanyl	10	50
	O‐Desmethyltramadol	10	50
	Oxycodone	10	100
	Oxymorphone	10	100
	Tramadol	10	50
Gabapentinoid	Gabapentin	50	500
	Pregabalin	50	500
Other sedative medication	Fluoxetine	10	50
	Diphenhydramine	10	50

### 2.3 Statistical Analysis

We included all samples testing positive for at least one of the drug groups defined in Table 1.

Detection rates for each drug group, defined as the number of samples testing positive for that group divided by the number of samples testing positive for at least one of the included drug groups were calculated and reported overall, by gender, and age group, with associated chi‐square test *p*‐values.

The five most frequent combinations for the co‐detection of two drug groups in the same sample were determined. Co‐detection rates for these combinations are calculated as the number of samples with the co‐detected pair divided by the number of samples testing positive for at least one drug group. Co‐detection rates are reported overall, and by gender, with associated chi‐square test *p*‐values.

LCA was performed on sample drug groups' binary test results. We estimated a series of models with increasing numbers of classes and compared key LCA fit statistics including the Akaike information criteria and Bayesian information criteria (BIC). We visualised these statistics to determine which number of classes best fit the data, with a preference for BIC over Akaike information criteria in making the final determination [20]. We also considered each class interpretation to ensure we were not overestimating the number of classes present in the population or identifying unstable classes. A latent class was attributed to each sample as the class with the highest posterior probability. The final model entropy, size and percentage of each class are reported.

Descriptive statistics by latent class were calculated. We report the proportion of men, the median and interquartile range (IQR) for age at the time of sampling, as well as the percentage of samples with more than one drug group detected and the average number of drug groups detected per sample in each class. *P*‐values for chi‐square or Kruskal–Wallis tests are provided as appropriate.

## 3 Results

Between 2019 and 2020 toxicological analysis was conducted on a total of 6161 samples (89% blood/11% urine), collected from 5681 drivers. Fifty‐four samples were excluded due to missing age/gender or age < 15 years (*n* = 18), resulting in 6107 samples. The large majority of toxicology samples were from men (88.8%), and the median [IQR] age at sampling was 30 years [24, 25, 26, 27, 28, 29, 30, 31, 32, 33, 34, 35, 36, 37, 38]. Among all samples, 4856 (79.5%) tested positive for at least one of the following drug groups: amphetamine, alprazolam, NPS benzodiazepine, other benzodiazepine, cannabis, cocaine, gabapentinoids, ketamine, methamphetamine, heroin, codeine, methadone (EDDP), other opioid, other sedating drug or z‐drugs. Two or more drug groups were co‐detected in 2081 (34.1%) samples.

### 3.1 Detection Rates

Detection rates by drug group are presented in Table 2, overall, by gender, and by age group. Among positive samples, the most commonly detected drug group was cannabis (70.1%) followed by cocaine (40.2%) and other benzodiazepines (15.7%). Cannabis detection was more common in men (72% vs. 51% in women, *p* < 0.001), and was gradually decreasing from younger (79% in 16–24 years) to older (45% in 45+ years) age groups. The detection of cocaine was also higher in men (41% vs. 36% in women, *p* = 0.049), and peaked in age groups 25–34 years (42.7%) and 35–44 years (43.7%). In relation to other drug groups, women had higher detection rates of non‐NPS benzodiazepines, z‐drugs, gabapentinoids and opioids compared to men. The detection of benzodiazepines (excluding NPS benzodiazepines and alprazolam), z‐drugs, gabapentinoids, codeine, heroin, other opioids and other sedative medications was highest in those aged 45 and over.

**TABLE 2:** Detection rates of amphetamine, alprazolam, new psychoactive substances (NPS) benzodiazepine, other benzodiazepine, cannabis, cocaine, gabapentinoids, ketamine, methamphetamine, heroin, codeine, methadone, other opioid, other drowsy medication and z‐drug among driver samples positive for at least one drug group in 2019–2020 (*N* = 4856) overall, by sex and age class.

	Sex			Age class					Total samples, *N* = 4856
	Female, *n* = 454	Male, *n* = 4402	*p* ^a^	16–24, *n* = 1343	25–34, *n* = 2067	35–44, *n* = 1044	45+, *n* = 402	*p* ^a^	
Amphetamine	18 (4.0%)	180 (4.1%)	0.899	37 (2.8%)	88 (4.3%)	62 (5.9%)	11 (2.7%)	0.001	198 (4.1%)
Benzodiazepine NPS	15 (3.3%)	127 (2.9%)	0.614	27 (2.0%)	61 (3.0%)	41 (3.9%)	13 (3.2%)	0.051	142 (2.9%)
Alprazolam	82 (18.1%)	444 (10.1%)	< 0.001	87 (6.5%)	253 (12.2%)	148 (14.2%)	38 (9.5%)	< 0.001	526 (10.8%)
Other benzodiazepine	118 (26.0%)	643 (14.6%)	< 0.001	111 (8.3%)	305 (14.8%)	245 (23.5%)	100 (24.9%)	< 0.001	761 (15.7%)
Cannabis	232 (51.1%)	3174 (72.1%)	< 0.001	1057 (78.7%)	1535 (74.3%)	633 (60.6%)	181 (45.0%)	< 0.001	3406 (70.1%)
Cocaine	163 (35.9%)	1790 (40.7%)	0.049	498 (37.1%)	882 (42.7%)	456 (43.7%)	117 (29.1%)	< 0.001	1953 (40.2%)
Gabapentinoid	56 (12.3%)	193 (4.4%)	< 0.001	41 (3.1%)	85 (4.1%)	75 (7.2%)	48 (11.9%)	< 0.001	249 (5.1%)
Ketamine	7 (1.5%)	59 (1.3%)	0.724	31 (2.3%)	20 (1.0%)	6 (0.6%)	9 (2.2%)	< 0.001	66 (1.4%)
Methamphetamine	13 (2.9%)	134 (3.0%)	0.831	44 (3.3%)	63 (3.1%)	34 (3.3%)	6 (1.5%)	0.296	147 (3.0%)
Codeine	46 (10.1%)	193 (4.4%)	< 0.001	18 (1.3%)	85 (4.1%)	96 (9.2%)	40 (10.0%)	< 0.001	239 (4.9%)
Heroin	63 (13.9%)	231 (5.3%)	< 0.001	19 (1.4%)	109 (5.3%)	105 (10.1%)	61 (15.2%)	< 0.001	294 (6.1%)
Methadone	50 (11.0%)	242 (5.5%)	< 0.001	3 (0.2%)	91 (4.4%)	150 (14.4%)	48 (11.9%)	< 0.001	292 (6.0%)
Other opioid	30 (6.6%)	100 (2.3%)	< 0.001	8 (0.6%)	55 (2.7%)	33 (3.2%)	34 (8.5%)	< 0.001	130 (2.7%)
Other sedative medication	32 (7.1%)	35 (0.8%)	< 0.001	4 (0.3%)	22 (1.1%)	19 (1.8%)	22 (5.5%)	< 0.001	67 (1.4%)
Z‐drug	50 (11.0%)	143 (3.3%)	< 0.001	22 (1.6%)	59 (2.9%)	70 (6.7%)	42 (10.5%)	< 0.001	193 (4.0%)

^a^ Chi‐square test.

The five most commonly co‐detected drug groups are presented in Table 3, overall and by gender. Cocaine and cannabis were co‐detected in 19.5% of samples positive for at least one drug group, followed by cocaine and other benzodiazepines (8.2%), cannabis and other benzodiazepines (8.1%), cannabis and alprazolam (6.2%), cocaine and alprazolam (5.6%). Cocaine and cannabis were more often co‐detected in men (20% vs. 14% in women, *p* = 0.002) whereas cocaine and alprazolam were more co‐prevalent in women (8% vs. 5% in men, *p* = 0.045).

**TABLE 3:** Co‐detection rates of cocaine and cannabis; cocaine and other benzodiazepine; cannabis and other benzodiazepine; cannabis and alprazolam; cocaine and alprazolam among driver samples positive for at least one drug group in 2019–2020 (*N* = 4856) in 2019–2020, overall and by sex.

	Sex			Total samples, *N* = 4856
	Female	Male	*p* ^a^	
Cocaine and cannabis	64 (14.1%)	885 (20.1%)	0.002	949 (19.5%)
Cocaine and other benzodiazepine	42 (9.3%)	354 (8.0%)	0.37	396 (8.2%)
Cannabis and other benzodiazepine	41 (9.0%)	354 (8.0%)	0.463	395 (8.1%)
Cannabis and alprazolam	30 (6.6%)	273 (6.2%)	0.733	303 (6.2%)
Cocaine and alprazolam	35 (7.7%)	239 (5.4%)	0.045	274 (5.6%)

^a^ Chi‐square test.

### 3.2 Latent Class Analysis

**FIGURE 1:**
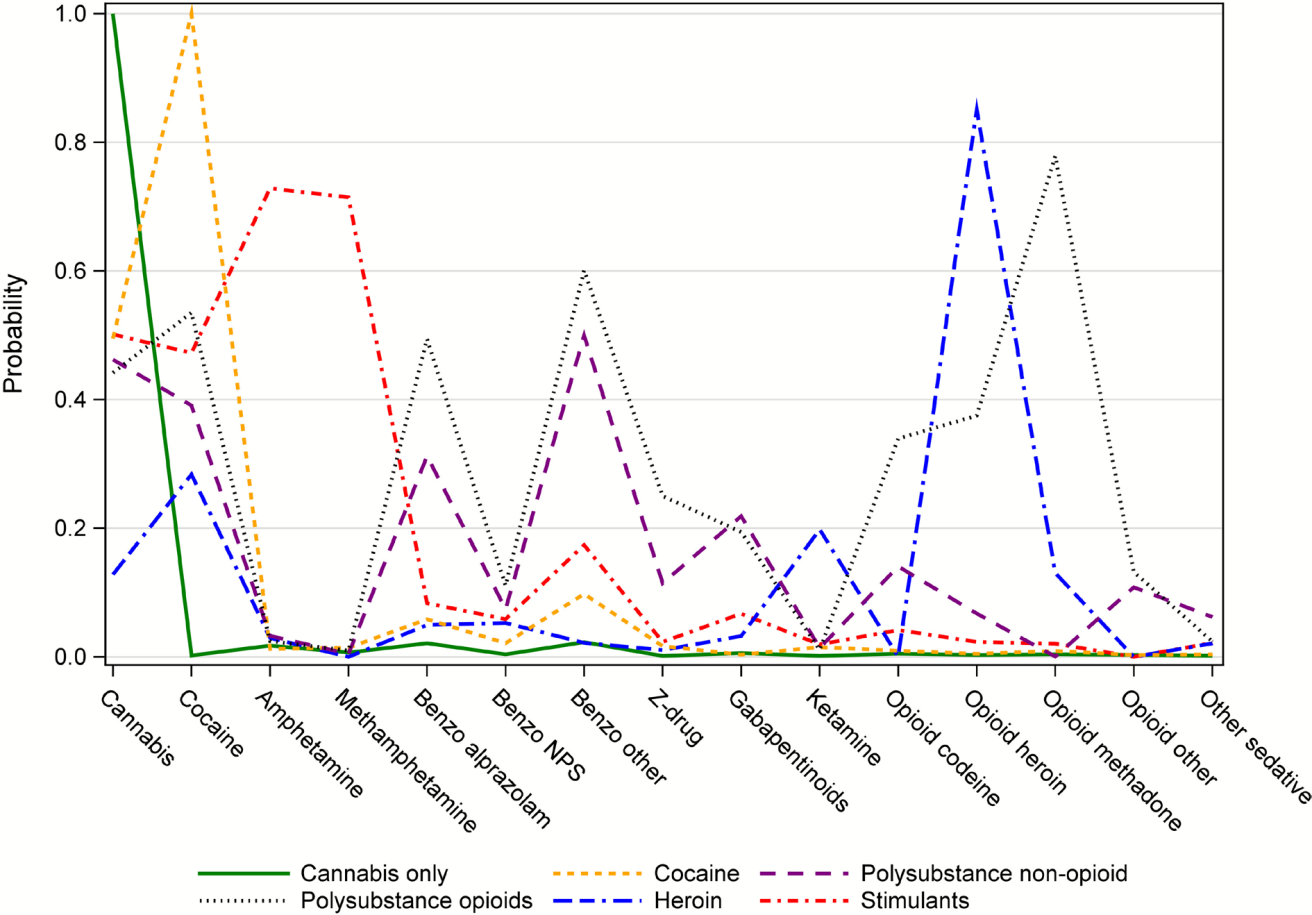
Conditional probabilities of detection for each substance group indicator by latent class.

The fit statistics for the latent class models are displayed in Table 4. We selected a 6‐class model based on the lowest BIC and interpretability of the resulting classes. The conditional probabilities of each drug group indicator by latent class are displayed in Figure 1. Class sizes based on the highest posterior probability are presented in Table 5.

**TABLE 4:** Fit statistics for 2–7 classes latent class analysis models.

N classes	Log likelihood	AIC	BIC	Entropy
2	−17052.1	3795.7	3996.8	0.733
3	−16550.4	2824.3	3129.3	0.841
4	−16345.7	2446.8	2855.6	0.861
5	−16247.4	2282.3	2794.9	0.876
**6**	**−16169.8**	**2159.0**	**2775.3**	**0.851**
7	−16111.6	2074.6	2794.8	0.846

*Note:* Selected model in bold.

Abbreviations: AIC, Akaike information criteria; BIC, Bayesian information criteria.

**TABLE 5:** Size and demographic characteristics of the six latent classes.

	Latent class						*p* ^a^	Total samples, *N* = 4856
	Cannabis only	Cocaine	Polydrug non‐opioid	Polydrug opioids	Heroin	Stimulants		
Total								
*N*	2259	1512	573	267	126	119		
%	46.5%	31.1%	11.8%	5.5%	2.6%	2.5%		
Percentage of males	93.6%	93.0%	79.1%	82.0%	78.6%	93.3%	< 0.001	90.7%
Age at time of sampling								
Median [Q1–Q3]	28 [23–34]	29 [24–34]	34 [27–42]	38 [33–42]	36.5 [29–46]	30 [24–36]	< 0.001	29 [24–36]
Percentage with more than one drug group detected	12.4%	61.4%	75.6%	99.6%	59.5%	83.2%	< 0.001	42.9%
Number of drug groups detected								
Mean	1.12	1.77	2.79	4.66	1.79	3.16	< 0.001	1.78
Median [Q1–Q3]	1 [1–1]	2 [1–2]	3 [2–4]	4 [4–6]	2 [1–2]	3 [2–4]		1 [1–2]

^a^ Kruskal–Wallis or chi‐square test as appropriate.

The largest class (46.5%) is characterised by the detection of cannabis with no other drug involved (class name: Cannabis only). High levels of cocaine and moderate levels of cannabis detection defined the second class (31.1%) (class name: Cocaine). The third class (11.8%) is characterised by moderate detection of cocaine and cannabis, as well as non‐NPS benzodiazepines, including alprazolam, and low detection levels for opioid class drugs (class name: Polydrug non‐opioid). The fourth class (5.5%) is characterised by the detection of opioids, including methadone, heroin and codeine, with additional drugs detected at a moderate level, including cannabis, cocaine, and benzodiazepines (class name: Polydrug opioids). The fifth class (2.6%) is a small group containing samples with high heroin detection, and minor detection of cocaine and ketamine (class name: Heroin). The last class (2.5%) was well defined by a high probability of detection of amphetamine/methamphetamine (vs. low detection of these drugs in other latent classes), associated with moderate detection of cannabis and cocaine, and low detection of other drugs (class name: Stimulants).

The demographic characteristics of each latent class as well as the average number of drug groups detected are presented in Table 4. The median age was similar at just below 30 years in the cannabis only, cocaine and stimulant classes, with the percentage of males close to 93%. In contrast, heroin, opioid and non‐opioid polydrug classes were older (34–38 years) and had a relatively lower proportion of men (79%–82%). The average number of drug groups detected was highest in the polydrug opioids class (4.7 out of 15 drug groups), followed by stimulants (3.2), polydrug non‐opioid (2.8), cocaine (1.8), heroin (1.8) and cannabis only (1.1).

## 4 Discussion

### 4.1 Main Results in Context

In this study of toxicological samples from people suspected of driving under the influence of an intoxicant, over 90% of positive samples were collected from men, similar to data previously reported [30]. The most commonly detected drugs were cannabis, followed by cocaine, benzodiazepines other than alprazolam, and alprazolam. This is consistent with road toxicology findings in Europe [31] and Ireland [30], and reflects cannabis being the most commonly reported illicit drug in Ireland (5.9% prevalence for last year drug consumption in the general adult population) [32]. Differences in cannabis and cocaine detection rates by gender are in line with international literature with higher rates consistently observed in male compared to female drivers [33]. In this study, prescription drugs including benzodiazepines, gabapentinoids and opioids were more likely to be detected in women, whereas no difference was noted in the review mentioned above for opioids and benzodiazepines/z‐drugs [33]. However, this should be interpreted with caution as our sample is subject to selection bias and is not representative of the general driving population.

While research mostly reports rates of drug use independently among drivers, few studies have investigated polydrug use patterns in drivers. A LCA of a national random sample of multiple‐substance‐using drivers in the United States in 2007 (*n* = 250) identified four classes: ‘cocaine and cannabis’, ‘polysubstance’, ‘cannabis and opioids’ and ‘prescription opioids and benzodiazepines’ [24]. However, methodological differences between the studies limit comparisons, as Scherer et al. only included samples with two or more drugs detected, and had a much smaller sample size. In the work presented here six latent classes have been identified based on drug detection patterns, characterised by varying degrees of single and polydrug detection, indicating that people driving after using drugs are not a homogenous group.

The cannabis‐only class was the youngest (median [IQR] 28 [23‐34] years), consistent with the prevalence of cannabis use being highest in adolescents and younger adults in the general population [32] as well as in younger drivers [34]. With moderate to high probability of detection of both cocaine and cannabis, the cocaine class identified here is similar to the cocaine and cannabis latent class identified in US drivers [24, 25], consistently relatively young (median [IQR] here 29 [24‐34] years) and with a high male:female ratio compared to other classes. The frequent co‐detection of cannabis with cocaine is consistent with findings from a meta‐analysis where the pooled prevalence of simultaneous cannabis use among cocaine users was 38% [35]. An increased RTC risk was found for this latent class in US drivers [25], consistent with epidemiological evidence [36]. Cocaine use [32], including in drivers under the influence of drugs [10] has been increasing in Ireland over the last decade, suggesting a need for countermeasures to reduce use in the general population and by extension in drivers. The polydrug non‐opioid class presents a wide age range (interquartile range = 15 years), which could support the hypothesis of a mixed group, inclusive of drivers receiving prescribed benzodiazepines and recreational non‐opioid drug users. The polydrug opioid class is characterised by a relatively older age compared to other classes (median 38 years). This is compatible with estimates for people seeking treatment for problem opioid use (40% of those seeking treatment for heroin or other opioids as main problem drug were aged 35–44 in 2020) [37] in Ireland. This class also had the highest number of drug groups detected (4.7), with high levels of benzodiazepines, cocaine and cannabis detection, reflecting toxicology findings from a large centre providing opioid agonist therapy for opioid use disorder in Dublin [38]. The stimulants class was relatively small (2.5%), reflecting a low prevalence of amphetamine‐like stimulants use in Ireland [32]. International evidence indicates that methamphetamines were sometimes used by professional drivers for their stimulant effect [39], however it is not known whether this is the case in our data. The heroin class reflects primarily heroin use in isolation or with one other drug group (average 1.8 drugs detected) with the detection of cocaine and ketamine at low to moderate levels. The age and gender profile was close to the polydrug opioid class, consistent with high‐risk opioid users in Ireland [37].

### 4.2 Implications

While the study design did not allow for the estimation of prevalence rates, characterising subpopulations of drug drivers can inform policy and further research, as well as the design and implementation of relevant interventions. It was suggested that pooling polydrug use indistinctively may not be pertinent, as some polydrug user profiles appear at greater risk for RTC involvement and alcohol consumption than others [25].

We note that the levels of cannabis detection were moderate to high in five out of the six latent classes. This does not necessarily imply acute intoxication, as there is a long detection window for cannabis use, up to several weeks for heavy use [40]. However, it demonstrates substantial use across most subpopulations identified. Cannabis is commonly consumed by people who use other drugs [35]. This may be of relevance as even non‐intoxicated cannabis users appear to have poorer driving performance than controls [41].

Regarding prescribed and non‐prescribed opioid use and driving safety, a review concluded that patients receiving long‐term analgesic opioids on pharmacologically stable doses of opioids are able to drive under conditions of: no co‐prescriptions or other psychoactive substance use (alcohol and illicit drugs); well‐controlled pain; no sleep disorder or daytime somnolence; and no other diagnosable psychiatric condition (e.g., significant depression or anxiety disorder) [42]. However, dual diagnoses of current depression (36%) and anxiety (29%) are highly prevalent in people with opioid use disorder [43]. Therefore caution should be exercised and authors of the former review recommend an individual evaluation of opioid agonist therapy patients' driving impairment [44].

Interventions to reduce driving under the influence of drugs mainly revolve around legislation, increased detection, and education [34]. In Ireland, all drivers are legally obligated to notify the National Driver Licence Service if they develop a medical condition that could affect their driving ability, including alcohol or drug dependence, and their licence is withheld until stable on treatment [45]. In addition to legal and financial penalties, evidence suggests that individuals need to believe there is a substantial probability of detection to refrain from engaging in prohibited behaviours. Regular and well‐targeted drug testing campaigns including roadside drug testing [46] may contribute to deterring drug driving. Furthermore, educational interventions could be extended and adapted to populations at risk of drug driving, including cannabis users, club/dance drug users, illicit opioid users and professional drivers. However, qualitative evidence suggests that strategies like media campaigns or improved detection methods are unlikely to significantly impact problematic drug users' behaviour [47], underlining the need to develop effective preventive strategies for this group. Incorporating drug driving prevention within drug treatment programmes was also suggested as a realistic approach [47]. Such harm‐reduction interventions could be aimed at medical staff and opioid agonist therapy patients, as well as people who use drugs [48]. Lastly, infrastructure interventions such as public transport available in locations/events where people are likely to consume drugs, as motivations for driving under the influence of drugs include the need to get home [49], may also be useful.

### 4.3 Strengths and Limitations

This study benefits from national coverage of all drivers' samples analysed for toxicology in 2019–2020 in Ireland, providing an exhaustive cross‐sectional representation of people suspected of driving under the influence of an intoxicant. Routine LC–MS drug screening techniques allow for the detection of a wide range of drugs, providing a detailed toxicological profile of drivers. The large size of the dataset with 4856 samples allowed identifying stable, small size classes. Finally, latent classes were characterised with gender and age covariates, providing important information for developing prevention and enforcement efforts [33].

Nonetheless, this study does have some limitations. Firstly, the population is limited to samples from drivers showing signs of intoxication or a positive oral fluid PDT and with alcohol levels below a specified threshold. This may lead to both overestimation (due to observed impairment, or positive PDT) and underestimation (due to the alcohol threshold) of the observed drug detection rates compared to the general driving population. However, the patterns identified remain relevant within this select population. Secondly, the concurrent use of co‐detected drugs cannot be established, as some drugs may remain detectable for longer than others. Positive findings may represent a single episode of use in the days prior to detection (e.g., benzodiazepines or cocaine) or heavy use that ceased more than several weeks ago (e.g., cannabis), and provide no information on intent [40]. In addition, it cannot be assessed from the data whether medications were prescribed or illicitly sourced, which can limit the interpretation of the latent classes. Thirdly, the analysis was conducted using aggregated drug groups. Due to the complexity of benzodiazepines metabolism the detection of only alprazolam could be separated from other common benzodiazepines, leading to a large group of “other benzodiazepines” medications. It is notable that in this other group diazepam and its metabolites oxazepam and temazepam were the most prevalent. Assumptions were made regarding the detection of heroin by morphine only, as documented in prior publications [50], however this could lead to an overestimation of heroin detection. Finally, while age and gender provide useful insight into socio‐demographic profiles, limited access to covariates precludes further interpretation of the findings.

Future research should aim to characterise polydrug driver profiles further, with the view of informing both the need and the design of interventions. Characterisation such as type of vehicle, type of driver, driver prior road offences as well as harms including speeding offence, concurrent alcohol consumption and collision involvement and severity would make a significant contribution to the existing body of knowledge.

## 5 Conclusion

While research mostly reports rates of drug use among drivers independently, this study examines multiple drug detection patterns in people suspected of driving under the influence of a drug intoxicant in Ireland. We identified six latent classes based on drugs detected in biological samples, indicating that people driving under the influence of drugs are not a homogenous group. This study contributes to a better knowledge of drug and polydrug use within the context of road use in Ireland. Characterising subpopulations of people driving under the influence of drugs can inform further research, as well as the design and implementation of relevant interventions and policies, and should be further resourced and investigated.

## Author Contributions

Conceptualisation: L.D., G.C. Data curation: L.D., A.O.K., R.M. Formal Analysis: L.D. Funding acquisition: R.M., D.C., E.K., G.C. Investigation: L.D., R.M., G.C. Methodology: L.D. Project administration: G.C. Resources: R.M., D.C. Software: L.D. Supervision: G.C. Validation: A.O.K., R.M. Visualisation and writing – original draft: L.D. Writing – review and editing: L.D., A.O.K., R.M., D.C., E.K., G.C. Each author certifies that their contribution to this work meets the standards of the International Committee of Medical Journal Editors.

## Funding

This study was funded through the Health Research Board under the Secondary Data Analysis Projects SDAP‐2021‐009.

## Ethics Statement

The study has received approval from the RCSI Ethics Committee (REC202202020).

## Conflicts of Interest

The authors declare no conflicts of interest.

## Supporting information


**Appendix S1:** Supporting information.

## Data Availability

Research data are not shared.
